# Nano-sized carriers in gene therapy for renal fibrosis *in vivo*

**DOI:** 10.1080/20022727.2017.1331099

**Published:** 2017-06-13

**Authors:** Haruhisa Miyazawa, Keiji Hirai, Susumu Ookawara, Kenichi Ishibashi, Yoshiyuki Morishita

**Affiliations:** a Division of Nephrology, First Department of Integrated Medicine, Saitama Medical Center, Jichi Medical University, Saitama, Japan; b Department of Medical Physiology, Meiji Pharmaceutical University, Tokyo, Japan

**Keywords:** Renal fibrosis, gene therapy, nano-sized carriers, viral vector, non-viral vector

## Abstract

Renal fibrosis is the final common pathway leading to end-stage renal failure regardless of underlying initial nephropathies. No specific therapy has been established for renal fibrosis. Gene therapy is a promising strategy for the treatment of renal fibrosis. Nano-sized carriers including viral vectors and non-viral vectors have been shown to enhance the delivery and treatment effects of gene therapy for renal fibrosis *in vivo*. This review focuses on the mechanisms of renal fibrosis and the *in vivo* technologies and methodologies of nano-sized carriers in gene therapy for renal fibrosis.

**RESPONSIBLE EDITOR** Alexander Seifalian Director of Nanotechnology & Regenerative Medicine Ltd., The London BioScience Innovation Centre, London, UNITED KINGDOM

## 1. Introduction

Chronic kidney disease (CKD), defined as a decreasing glomerular filtration rate and/or the presence of histological or biochemical markers of kidney damage over 3 months, is a worldwide public health problem [1]. A recent meta-analysis reported that the global prevalence of CKD is estimated as 13.4% in general populations [2]. In the pathology of CKD, tubulointerstitial fibrosis, called renal fibrosis, is the final common pathway leading to end-stage renal disease regardless of initial nephropathies [3,4]. As no specific therapy has been established for renal fibrosis, the development of treatment options is crucial to improve the prognosis for CKD patients. Understanding the mechanisms of renal fibrosis enables targeting of the processes that take place to prevent them from occurring. Many different kinds of cells, such as immune, vascular endothelial, tubular epithelial, and fibroblast cells, have been considered to contribute to renal fibrosis *in vivo* [5–10]. Studying therapeutic approaches for the treatment of renal fibrosis *in vivo* is important. Gene therapy is a potentially promising strategy for the treatment of renal fibrosis *in vivo* because it can target molecules that were previously difficult to set as therapeutic targets using small molecules or antibodies. The development of nano-sized carriers including viral vectors and non-viral vectors has been shown to enhance the delivery and treatment effects of gene therapy for various diseases, including renal fibrosis *in vivo* [11–14].

This review focuses on the mechanisms of renal fibrosis and the *in vivo* technologies and methodologies of nano-sized carriers in gene therapy for renal fibrosis.

## 2. Mechanisms of renal fibrosis

**Figure 1.**
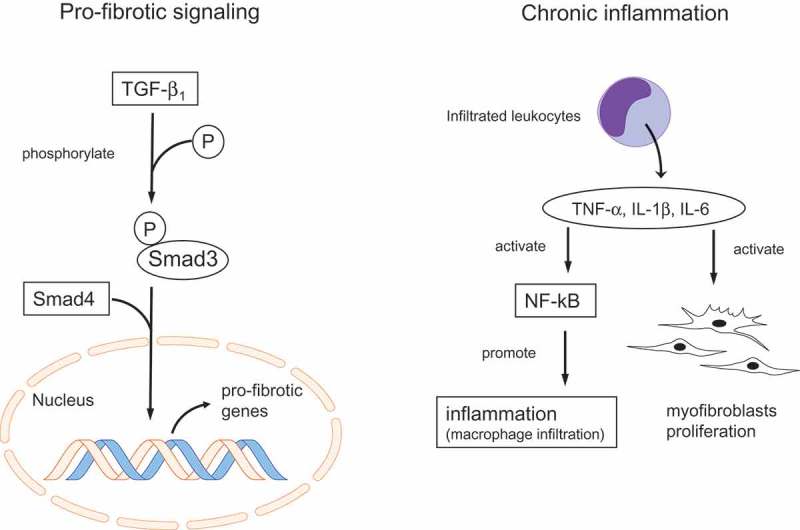
Mechanisms of renal fibrosis. TGF: transforming growth factor; Smads: small mothers against decapentaplegic; P: phosphorylation.

Renal fibrosis is pathologically characterized by the proliferation of myofibroblasts and the excessive accumulation of extracellular matrix components such as fibrotic collagen in the tubulointerstitial space [15]. Although the precise mechanisms of renal fibrosis have not been completely determined, activation of pro-fibrotic signaling pathways and chronic inflammation are thought to play central roles in renal fibrosis (Figure 1) [16–25].

### 2.1. Pro-fibrotic signaling

Numerous studies have demonstrated that transforming growth factor (TGF)-β_1_ and downstream intracellular proteins, known as small mothers against decapentaplegic (Smad), play a central role as a pro-fibrotic pathway in renal fibrosis (Figure 1) [16–22]. TGF-β_1_ receptor-activated Smad3 combines with a common Smad, called Smad4, and this complex is translocated into the nucleus where it binds to DNA elements to promote the transcription of various pro-fibrotic genes (Figure 1) [16–19]. Therapeutic agents that inhibit TGF-β_1_–Smad signaling have been shown to reduce extracellular matrix accumulation in the tubulointerstitial space, resulting in the inhibition of renal fibrosis [16,26,27].

### 2.2. Chronic inflammation

Unresolved inflammation characterized by leukocyte infiltration is considered to be a main factor contributing to renal fibrosis (Figure 1) [23–25,28,29]. Infiltrated leukocytes excrete various pro-fibrotic growth factors and cytokines such as tumor necrosis factor-alpha (TNF-α), interleukin (IL)-1β, and IL-6, which promote the proliferation of myofibroblasts (Figure 1) [29,30]. Levels of these cytokines in plasma have been shown to increase in patients with CKD [31,32]. These cytokines activate nuclear factor-kappa B (NF-κB), which is a major signaling pathway that activates and promotes macrophage infiltration, further increasing the inflammatory pathway (Figure 1) [33,34]. Inhibition of the NF-κB signaling pathway was shown to inhibit renal injury, whereas activation of this pathway was shown to promote renal injury, including renal fibrosis [33,34].

## 3. Viral and non-viral vectors for renal fibrosis *in vivo*

**Figure 2.**
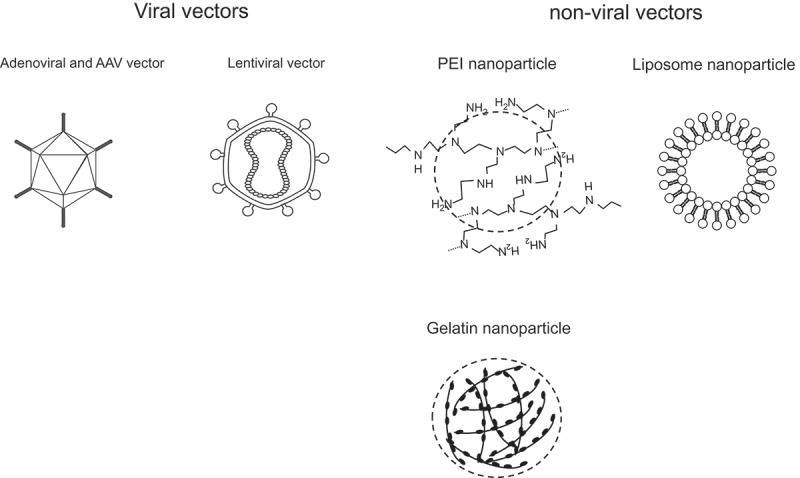
Viral and non-viral vectors for renal fibrosis *in vivo*. TNF: tumor necrosis factor; IL: interleukin; NF-κB: nuclear factor-kappa B.

Viral vectors and non-viral vectors have been studied as nano-sized carriers in gene therapy for renal fibrosis *in vivo* (Figure 2). These vectors enhance delivery and increase the efficiency of gene transduction into the tubulointerstitial space and enhance the anti-fibrotic effects of gene therapy *in vivo*. The categories of vectors, genes, administration routes, and the effects of renal fibrosis *in vivo* are summarized in Table 1.

**Table 1.** Gene therapies using viral and non-viral vectors for the treatment of renal fibrosis.

Vector		Gene	Administration route	Effects	Authors (year published)	Reference No.
Virus vectors	Adenovirus	TGF-β_1_ type II receptor 1-DNA	Intramuscle injection	Reduced fibrosis in both glomeruli and tubulointerstitial area	Kondo et al. (2008)	[35]
	Adenovirus	Runx2-DNA	Intraperitoneal injection	Attenuated TGF-β_1_-induced Smad3 phosphorylation and expression levels of α-smooth muscle actin and collagen I in the fibrotic kidney	Kim et al. (2013)	[36]
	Adenovirus	Decorin-DNA	Intravenous injection	Inhibited expression levels of TGF-β_1_ mRNA and protein and reduced fibrosis in the fibrotic kidney	Zhang et al. (2010)	[37]
	Adenovirus + (electroporation)	Smad7-DNA	Intrapelvic injection	Over-expressed Smad7 in the kidney and inhibited renal fibrosis	Terada et al. (2008)	[38]
	Adenovirus	IκB-α-DNA	Intravenous injection	Inhibited NF-κB activation by over-expression of IκB-α in the renal cortex and ameliorated tubulointerstitial injury	Takase et al. (2003)	[39]
	AAV	HGF-DNA	Intravenous injection	Attenuated tubulointerstitial fibrosis	Schievenbusch et al. (2010)	[40]
	AAV	IL-10-DNA	Intravenous injection	Over-expressed IL-10 in plasma and inhibited renal fibrosis by inhibiting infiltration of T lymphocytes and macrophages	Mu et al. (2005)	[41]
	AAV	SOCS-DNA	Intrarenal injection	Over-expressed SOCS2 in the kidney and inhibited renal fibrosis and inflammation in the fibrotic kidney	Zhou et al. (2007)	[42]
	AAV	Adrenomedullin-DNA	Intravenous injection	Decreased blood pressure and ameriolated glomerular sclerosis, tubular injuries, and protein casts in the kidney	Wang et al. (2001)	[43]
	AAV	eNOS-DNA	Intravenous injection	Prevented an increase in blood pressure and proteinuria and reduced glomerular and tubular injury	Savard et al. (2012)	[44]
	AAV	Klotho-DNA	Intravenous injection	Prevented the progression of renal hypertrophy and fibrosis	Deng et al. (2015)	[45]
	AAV	CYP2J2-DNA	Intravenous injection	Decreased blood pressure and proteinuria and ameliorated renal fibrosis	Zhao et al. (2013)	[46]
	AAV	ATIIR1-DNA	Intravenous injection	Decreased blood pressure and inhibited renal injury	Li et al. (2007)	[47]
	Lentivirus	prohibitin-DNA	Intraperitoneal injection	Ameliorated renal fibrosis	Zhou et al. (20013)	[48]
Non-viral vectors	PEI	PAX2-siRNA	Renal capsule injection	Inhibited 51% of PAX2 mRNA and 81% of PAX2 protein and ameliorated renal fibrosis	Li et al. (2012)	[49]
	PEI	microRNA-146a-mimic	Intravenous injection	Inhibited renal fibrosis by inhibiting TGF-β_1_ and NF-κB signaling pathways	Morishita et al. (2015)	[50]
	Liposome	FITC-labeled ODN	Retrograde injection through the ureter	Delivered FITC-labeled ODN into the nuclei of renal interstitial cells	Tsujiw et al. (2000)	[51]
	Cationic gelatin	TBR-siRNA	Retrograde injection through the ureter	Inhibited TBR expression and ameliorated renal fibrosis	Kushibiki et al. (2006)	[52]
	Cationic gelatin	HSP47-siRNA	Retrograde injection through the ureter	Inhibited HSP47 expression and diminished renal fibrosis	Xia et al. (2008)	[53]
	Cationic gelatin	MMP-DNA	Intraperitoneal injection	Inhibited renal fibrosis	Aoyama et al. (2003)	[54]

AAV: adeno-associated viral; TGF-β_1_: transforming growth factor-β_1_; Runx2: runt-related transcription factor 2; Smad7: small mothers against decapentaplegic 7; IκB-α: I-kappa-B-alpha; NF-κB: nuclear factor-kappa B; SOCS: suppressor of cytokine signaling; eNOS: endothelial nitric oxide synthase; ATIIR1: angiotensin II receptor 1; HGF: hepatocyte growth factor; IL-10: interleukin-10; PAX2: paired box 2; FITC: fluorescein isothiocyanate; ODN: oligodeoxynucleotides; TBR: transforming growth factor-β_1_ receptor; HSP47: heat shock protein 47; MMP: matrix metalloprotease.

### 3.1. Viral vectors

Adenovirus and adenovirus-associated virus (AAV) vectors are the most widely used viral vectors in gene therapy for renal fibrosis *in vivo*, while only a few studies have reported on the role of lentiviral vectors. Many studies have targeted inhibition of the TGF-β_1_–Smad signaling pathway for the treatment of renal fibrosis. Various administration routes (intramuscle, intravenous, intra-arterial, intraperitoneal, intrarenal, intraureter) have been used for the delivery of genes to the tubulointerstitial space of the kidney.

#### 3.1.1. Adenoviral and AAV vectors

Adenoviral vectors can deliver and transfect genes into both dividing and non-dividing cells [55]. Adenoviral vectors are double-stranded, non-enveloped DNA viral vectors, 70–90 nm in diameter, with a genome of 36–38 kb [55,56]. Adenoviral vectors designed for expression of TGF-β_1_ type II receptor, which is a competitive inhibitor of TGF-β_1_, were injected into hindlimb muscles of a mouse model of diabetic nephropathy [35]. Five weeks after administration, this gene therapy appeared to reduce fibrosis in both glomeruli and tubulointerstitial spaces [35]. Another study reported that adenoviral vectors designed for expression of runt-related transcription factor 2 attenuated TGF-β_1_-induced Smad3 phosphorylation, and reduced expression levels of α-smooth muscle actin (α-SMA) and collagen I in the kidney of unilateral ureteral obstruction (UUO) mice produced by unilateral ureteral ligation, which is a representative animal model of renal fibrosis [36,57]. The administration of adenoviral vectors designed for expression of decorin, which is an inhibitor of TGF-β_1_, was found to inhibit expression levels of TGF-β_1_ mRNA and protein and reduce fibrosis in the kidney of a rat model of diabetic nephropathy [37]. A study has reported on gene therapy that combined adenoviral vectors delivery and *in vivo* electroporation to deliver genes to the kidney [38]. In that study, adenoviral vectors designed for expression of Smad7, which is an antagonist of TGF-β_1_–Smad signaling, were injected into the pelvic space of a rat model of renal fibrosis induced by UUO [38]. Then, electroporation was performed on the kidneys. Ten electric pulses (50 m/s duration at 100 V) at a rate of 1 pulse/s were administrated to the kidney by a pair of electrode disks rigged on the tips of tweezers [38]. That delivery system significantly over-expressed Smad7 in the kidney and inhibited renal fibrosis [38]. Delivery of genes with adenoviral vectors to the kidney to modulate inflammatory signals and cytokines has been reported to inhibit renal fibrosis *in vivo* [39–42]. Adenoviral vectors designed for expression of I-kappa-B-alpha (IκB-α), which is an inhibitor of NF-κB, were found to inhibit NF-κB activation by over-expression of IκB-α in the renal cortex and ameliorate tubulointerstitial injury characterized by fibrosis and infiltration of mononuclear cells in a rat model of renal fibrosis [39]. Although adenoviral vectors have shown successful gene delivery to the kidney for the treatment of renal fibrosis, there is room for improvement as a carrier of gene therapy. The exogenous genes delivered using adenoviral vectors do not integrate into the host genome and do not replicate during cell division, resulting in short periods of gene expression of the targeted cells [55]. To overcome this drawback of the adenoviral vector, AAV vectors have been developed [58]. AAV vectors are single-stranded, non-enveloped DNA viral vectors, 18–26 nm in diameter, with a genome of 4–5 kb [56,58,59]. AAV vectors can also transfect genes into both dividing and non-dividing cells, and may incorporate genes into the host genome. Therefore, the duration of gene expression delivered using AAV vectors is considered to be longer compared with that delivered by adenoviral vectors [58]. AAV vectors were shown to have a low immunogenicity compared with adenoviral vectors [58]. AAV vectors serotype 9, designed for expression of hepatocyte growth factor (HGF), which is an anti-fibrotic cytokine, showed remarkable attenuation of tubulointerstitial fibrosis in a mouse model of renal fibrosis [40]. The administration of an AAV vector designed for expression of IL-10, which is an anti-inflammatory cytokine, was shown to over-express IL-10 in plasma and inhibit renal fibrosis by inhibiting infiltration of T lymphocytes and macrophages in a rat model of renal fibrosis [41]. AAV vectors designed for expression of suppressor of cytokine signaling (SOCS)2 over-expressed SOCS2 in the kidney and inhibited renal fibrosis and inflammation in a rat model of diabetic nephropathy [42]. The inhibitory effects on renal fibrosis by modulation of expression of the following genes by exogenous genes delivered with AAV vectors have been reported: adrenomedullin [43]; endothelial nitric oxide synthase [44]; Klotho [45]; cytochrome P450 2J2 [46]; and angiotensin II receptor 1 [47].

#### 3.1.2. Lentiviral vectors

Lentiviral vectors can transfect genes into both dividing cells and non-dividing cells and may incorporate genes into the host genome [60]. The lentiviral vectors are enveloped, single-stranded RNA viral vectors, 80–130 nm in diameter, with a genome of 8–9 kb [60]. Since lentiviral vectors are a recent development following on from adenoviral and AAV vectors, few studies have reported on lentiviral vectors as carriers of gene therapy for renal fibrosis *in vivo*. However, increasing interest in lentiviral vectors indicates the rise of a new field of research in lentiviral vectors for gene therapy of renal fibrosis [48,61]. Lentiviral vectors designed for expression of prohibitin, which is a pleiotropic protein for cellular proliferation, apoptosis, transcription, and mitochondria protein folding, were shown to ameliorate renal fibrosis in a mouse model of renal fibrosis induced by UUO [48]. HIV-derived lentiviral vectors designed for expression of type II TGF-β receptor (TBRII) were reported to attenuate renal fibrosis better, as estimated by expression levels of extracellular matrix synthesis, such as fibronectin and collagen III, and expression of α-SMA in both cultured renal epithelial cells and renal fibroblasts compared with non-lentiviral constructs [61]. However, the treatment effects on renal fibrosis using that lentiviral vector designed for the expression of type TBRII have not been investigated *in vivo*.

### 3.2. Non-viral vectors

There are several non-viral vectors that can deliver genes to the kidney (Figure 2). Polyethylenimine (PEI) nanoparticles, liposome nanoparticles, and cationic gelatin nanoparticles have been demonstrated to show effective delivery of genes to the kidney and have promising treatment effects in animal models of renal fibrosis [49–54].

#### 3.2.1. PEI nanoparticles

PEI is a polymer, 50–100 nm in diameter, that is considered the preferable material for the preparation of non-viral vectors in terms of long-term safety and biocompatibility [62,63]. Small interfering RNA (siRNA) targeted paired box2 (PAX2) was reported to be delivered to the kidney with PEI nanoparticles via an intrarenal capsule injection in a mouse model of renal fibrosis induced by UUO [49]. PAX2–siRNA–PEI nanoparticles inhibited PAX2 mRNA and PAX2 protein in the kidney, and ameliorated renal fibrosis [49]. Delivery of microRNA-146a mimic with PEI nanoparticles was reported to over-express microRNA-146a in the fibrotic kidney induced by UUO, and inhibited renal fibrosis by inhibiting TGF-β_1_ and NF-κB signaling pathways *in vivo* [49–54].

#### 3.2.2. Liposome nanoparticles

Liposome nanoparticles, 100–150 nm in diameter, comprise phospholipids and cholesterol, which are the main components of the cell membrane, and therefore show high biocompatibility [64]. Despite these advantages of liposome nanoparticles, few studies have reported on the validity of using liposome nanoparticles for gene delivery in the treatment of renal fibrosis *in vivo*. One study has reported that artificial viral envelope-type hemagglutinating virus of Japan (HVJ) liposome nanoparticles could deliver fluorescein isothiocyanate-labeled phosphorothioate non-targeted oligodeoxynucleotides to the nuclei of renal interstitial cells 10 min after transfection by retrograde injection through the ureter [51]. However, treatment effects of genes delivered with HVJ liposome nanoparticles for renal fibrosis have not been investigated. These results suggest that liposome nanoparticles may quickly deliver genes to the kidney, and may be promising in gene therapy for renal fibrosis.

#### 3.2.3. Gelatin nanoparticles

Gelatin is a protein derived from collagen [65]. Cationic gelatin nanoparticles, 100–300 nm in diameter, which are produced by chemically introducing cations such as thylenediamine, putrescine, spermidine, or spermine to the carboxyl group of gelatin [66], have been used in gene therapy for renal fibrosis *in vivo* [52–54]. Plasmid DNA designed for the expression of TGF-β receptor (TBR) siRNA with cationized gelatin nanoparticles was administrated in the fibrotic kidney induced by UUO [52]. These nanoparticles were shown to inhibit TBR expression and ameliorated fibrotic changes in the fibrotic kidney compared with naked plasmid DNA designed for the expression of TBR siRNA injection [52]. Heat shock protein 47 (HSP47) siRNA with cationized gelatin nanoparticles was shown to knock down HSP47 expression and diminish renal fibrosis in a mouse model of renal fibrosis induced by UUO [53]. Cationic gelatin nanoparticles incorporating plasmid DNA expressing matrix metalloprotease was shown to prevent renal fibrosis in a mouse model of diabetic nephropathy produced by intraperitoneal injection of streptozotocin, which causes damage to the pancreas and results in diabetic nephropathy [54,67].

## 4. Other methods of gene therapy for renal fibrosis *in vivo*

Electroporation and ultrasound methods are reported to deliver genes effectively to the kidney, and treatment effects have been demonstrated in renal fibrosis *in vivo* [68,69].

### 4.1. Electroporation

Electroporation is a transfection technique in which electric pulse waves are applied to target cells, creating micro-holes in the cell membrane to allow for the passage of exogenous genes into the cell. Plasmid DNA expressing HGF was injected into the tibialis anterior muscles and six electric pulses at 100 V were delivered by a stainless steel tweezer electrode placed in a transverse orientation relative to the muscle fiber. After transfection, plasma HGF levels increased and renal fibrosis was inhibited in a rat model of the fibrotic kidney induced by 5/6 nephrectomy [68].

### 4.2. Ultrasound

Plasmid DNA expressing short hairpin RNA (shRNA) of connective tissue growth factor (CTGF) was loaded on to the surface of a cationic microbubble [69]. The plasmid-carrying microbubbles were then administrated intravenously to mice and ultrasound was applied locally to the kidney treated with ureteral obstruction. This method exhibited reduced mRNA and protein levels of CTGF and inhibited fibrotic changes in the fibrotic kidney induced by UUO [69]. Another study reported that delivery of plasmid DNA expressing shRNA of microRNA-433 to the kidney by ultrasound microbubble-mediated gene transfer suppressed the induction and progression of renal fibrosis in the fibrotic kidney induced by UUO [15].

## 5. Summary

Nano-sized carriers such as viral and non-viral vectors in gene therapy for renal fibrosis *in vivo* have been developed. However, their long-term efficacy, side effects including toxicity and unexpected genomic DNA alternations, and their effects on other organs have not been fully investigated and require further study. Studies are also needed to develop nano-sized carriers that can exclusively deliver genes to the kidney.
